# Self-management of geriatric syndromes - an observational study

**DOI:** 10.1186/s12877-023-04442-8

**Published:** 2023-11-10

**Authors:** Tino Prell, Rebecca Wientzek, Aline Schönenberg

**Affiliations:** 1 Department of Geriatrics, Halle University Hospital, Ernst-Grube-Str. 40, 06120 Halle (Saale), Germany; 2 Department of Neurology, Jena University Hospital, Jena, Germany

**Keywords:** Self-management, Older adults, Geriatric syndromes, Self-efficacy

## Abstract

**Background:**

Self-management of health refers to various actions and decisions that impact health outcomes. To improve health, independence, and quality of life (QoL) while reducing healthcare utilization and costs, patients’ self-management abilities can be enhanced. However, disease-specific self-management interventions may not be applicable for older adults with multiple illnesses. Instead, focusing on prevalent geriatric syndromes, such as frailty, cognitive decline, pain, incontinence, or impaired mobility, may be more beneficial. To achieve this, a detailed understanding of the specific needs of the older population is crucial.

**Methods:**

Patients who are 70 years old or older will be chosen from four geriatric hospitals, which include both inpatient and outpatient facilities. At baseline, each participant will undergo a comprehensive geriatric evaluation and answer various questionnaires that focus on their current self-management abilities, self-efficacy, anxiety, aging perception, and QoL. Moreover, extensive data on the presence and impact of geriatric syndromes will be gathered. Three and six months after the initial evaluation, follow-up assessments will be conducted to identify any changes in participants’ health, independence in daily activities, geriatric syndromes, cognition and mood, QoL, and self-management.

**Discussion:**

The present investigation aims to assess the factors that may facilitate or impede self-management in older adults afflicted with geriatric syndromes. Instead of concentrating on particular diseases, this study will analyze the association between self-management and geriatric syndromes. The information obtained will contribute to clinical expertise on the self-management habits of older adults and their effects on their well-being, autonomy, and overall QoL, as well as provide insights into geriatric syndromes. This valuable knowledge will be crucial for developing personalised programs to enhance self-management among older adults.

**Trial registration:**

German Trial Register (Deutsches Register Klinischer Studien) DRKS00031016.

## Background

The rise in chronic and age-related illnesses is directly linked to demographic change. This is especially true for older adults who suffer from multiple conditions simultaneously, leading to frequent hospital visits and increased healthcare costs [1, 2]. This puts a strain on healthcare systems and makes it difficult to provide adequate care [3–5]. In addition to individual illnesses such as diabetes, osteoporosis, or Parkinson’s disease, older age is often characterised by overarching geriatric syndromes (also called *geriatric* giants), such as frailty, instability, falls, incontinence, depression, and cognitive decline [6–8]. These syndromes are often more relevant to individuals than a single illness. This is particularly true in the face of multimorbidity, where it is difficult to assign symptoms to a single illness [3, 6, 9]. Despite the significance of geriatric syndromes, they are often overlooked in clinical practice due to a lack of complete understanding, necessitating further research to comprehend their impact [10]. However, various studies have confirmed a high prevalence of geriatric syndromes, which are associated with poor health outcomes and increased healthcare utilization [6–8]. For example, recent data from Möller and colleagues (2022) surveying more than 6700 older adults show higher rates of hospitalization, longer hospital stays, more frequent outpatient contact and higher levels of polypharmacy in patients with geriatric syndromes [8]. Using the same dataset, Liang et al. (2018) identified a prevalence rate for having at least one geriatric syndrome of approximately 65% among patients aged 65 to 74 years; this number rose to almost 80% among those aged 75 to 84 years [11]. As the population continues to age and the relevance of geriatric syndromes increases, it can be assumed that the frequent healthcare utilization due to geriatric syndromes may overload healthcare systems. Hence, self-management becomes critical to reducing the burden on healthcare systems and promoting independent participation in daily life [1, 8, 12–14]. Indeed, poor health outcomes, increased healthcare costs, and adverse health events are associated with ineffective utilization of self-management strategies at home [14, 15]. As Udlis [16] put it, “people cannot *not* self-manage”, as every choice made, whether regarding activity, diet, social interaction, or decision making, is a type of self-management. Thus, supporting patients in *how* they manage their health is crucial, as it is not a question of “*if*” they self-manage. As a result, the World Health Organization designated self-management improvement as a critical strategy for combating chronic illnesses [17].

The study of self-management has been a frequented area of research, with many clinical intervention trials being carried out [14, 18]. Despite this, there has been no agreement on definitions, measurements, or interventions related to self-management [16, 19]. This lack of consensus has led to many different definitions being used in research, making it difficult to compare studies and make systematic progress [18]. Aside from the variations in definitions, self-management is considered a complex and individualised behaviour that covers various health-related, social, and emotional factors and aims to improve disease management in the long term [20]. This behaviour incorporates daily tasks performed to control or mitigate the impact of illnesses on health, social life and well-being [21]. Overall, as Barlow and colleagues (2002) put it, self-management describes “the individual’s ability to manage the symptoms, treatment, physical and psychosocial consequences and life style changes” [22], taking into consideration symptom monitoring and appropriate behavioural and emotional responses [22].

Previous research has identified several important factors that influence self-management, including self-efficacy [23, 24], perceived control, and attitudes toward health [25], social support [23, 26], cognition [26], and physical and mental health [26]. For example, Banerjee and colleagues (2022) assessed self-management in patients with chronic back pain and identified physical disability/activity, depression, and catastrophizing thoughts as barriers to self-management [27]. An Iranian study on patients with diabetes furthermore highlights the positive impact of social support on self-management behaviour [23]. Previous interventions to improve self-management are often disease-specific [28–30] and therefore not appropriate for the older population due to multimorbidity. For example, if a patient has 5 different diagnoses, managing diabetes as one of them will not be able to eradicate the impact of Parkinson’s disease, osteoporosis, hypertension, and depression. As Allegrante and Wells (2019) say, “managing a specific disease condition as opposed to managing the patient,” [14] is not equipped to deal with multimorbidity, geriatric syndromes, and continuous age-related life changes. Aging is accompanied by continuous changes in health, independence, and social life, and older adults are particularly vulnerable, both physically and mentally. Attitudes towards aging, such as the perception of control over aging processes and belief in improvement, must also be considered when assessing self-management, as these have been shown to influence health behaviour [31–33]. Therefore, many previous studies have not adequately addressed the specific needs of older adults. To fully comprehend and improve overall health outcomes, it is essential to consider the complex interplay of multiple systems, instead of concentrating on disease-specific factors [18]. Consequently, several reviews emphasize the importance of conducting more comprehensive research on self-management and its associated factors to develop effective interventions [16, 19, 34].

To fully understand self-management in the older patient population, we devised an in-depth quantitative, multicenter, and longitudinal data collection that encompasses the characteristics of the older population and focuses on a multitude of factors involved in self-management. Through this process, we aim to achieve a comprehensive understanding of older patients` self-management abilities challenges, and opportunities. As most older adults suffer from multimorbidity, and thus targeting individual illnesses is not feasible, we plan to explore various geriatric syndromes and their impact on health as potential self-management targets. For this purpose, comprehensive data on geriatric syndromes must be collected.

## Methods/design

This study is part of a larger project aimed at improving self-management through an intervention trial. The manuscript outlines the procedure for collecting baseline and follow-up data, which will serve as the foundation for the intervention. The first objective of this data collection is to determine the prevalence and impact of geriatric syndromes in order to identify intervention targets that are relevant to the older population. The second objective is to understand the current self-management behaviour and abilities of older adults, including predictors, burdens, and barriers. This will help us understand the link between self-management and health outcomes, and identify the variables that need to be fostered to enable older adults to self-manage geriatric syndromes. The study procedure is described in detail below.

### Settings and participants

Adults aged ≥ 80 years (or ≥ 70 years with multimorbidity) will be recruited from the geriatric inpatient wards and outpatient settings of four German hospitals included in the Center for Geriatrics in Southern Saxony-Anhalt (Zentrum für Altersmedizin im südlichen Sachsen-Anhalt, ZASSA). Written informed consent will be obtained from all patients or their representatives, and the study will be conducted in accordance with the Declaration of Helsinki and the Good Scientific Practice. The local ethics committee of Halle University Hospital approved this study. To avoid selection bias, all patients admitted to the wards during the data collection period will be screened for eligibility. Patients will be excluded from the study if they decline to participate, have acute delirium or severe dementia, or are unable to perform any activities of daily living (ADLs) on their own (complete dependence, e.g. due to being bedridden) according to the Barthel Index [35]. The follow-up assessments will take place 3 and 6 months after a patient is discharged from hospital. Data collection is expected to be completed by June 2025.

The sample size calculation is based on the following considerations: for the exploratory analysis of predictors of self-management using linear regression, a minimum of 8 patients per covariate is necessary [36]. Thus, we planned 10 patients per covariate included in the model. When considering approximately 25 covariates (see below), a sample size of 250 is assumed to be sufficient for our analysis. For the longitudinal survey, we consider changes in ADL as an outcome parameter. For the exploratory analysis of ADL changes in the linear regression, 171 participants are necessary to detect a significant effect with a moderate effect according to Cohen (adjusted R^2^ = 0.13). When expecting a decline in ADL of at least 1 point by follow-up, a sample size of 204 subjects is required [37, 38]. Assuming a dropout rate of 30% due to the advanced age of our patients, the final desired sample size is 265 patients.

### Variables and data collection

**Fig. 1:**
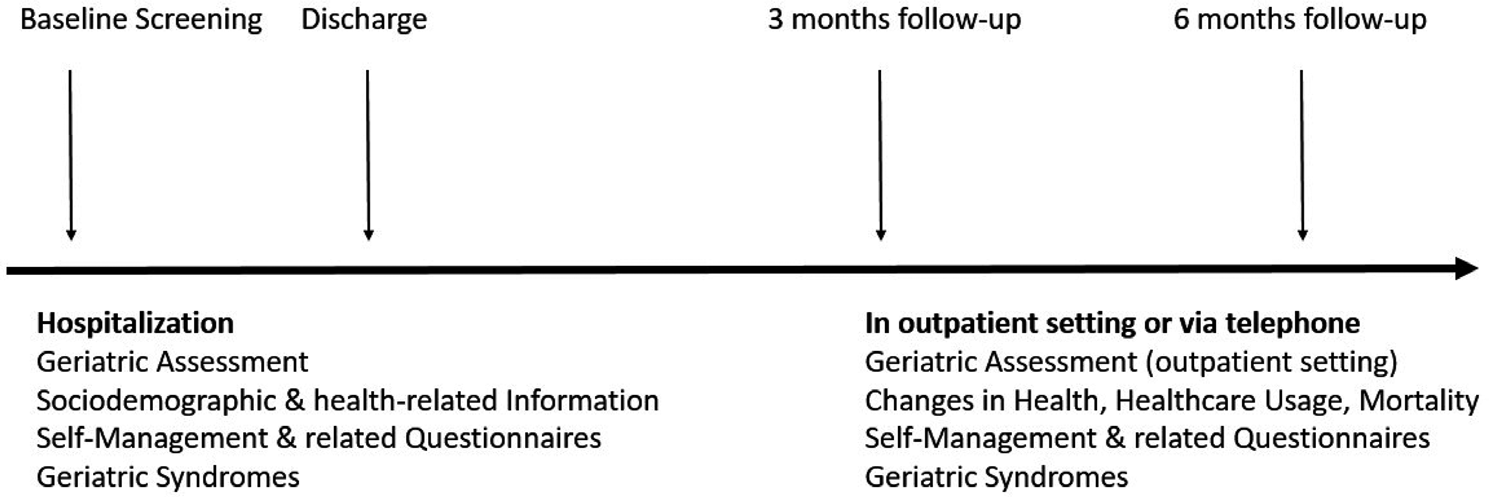
Study procedure

Data will be collected from patients at baseline during their hospital stay, and at two follow-up time points (3 and 6 months post-discharge, see Fig. 1 for an overview of the study procedure). Patients’ medical records as well as routine clinical assessments and additional questionnaires will be used to collect information on self-management and its related factors as well as geriatric syndromes.

During baseline data collection, each patient will undergo an encompassing geriatric assessment performed by medical staff on the wards. This includes the following assessments:

- Geriatric Screening for *functioning* as proposed by Lachs [39].
- Level of *independence* in ADLs and instrumental ADLs (iADLs) as assessed by the Barthel Index [35] and Blaylock Score [40]. The Blaylock Score assesses independent management of finances, medication, use of transportation, grocery shopping, and meal preparation.
- Level of *Loneliness* as assessed by the UCLA 3-Item Loneliness Scale [41].
- *Cognition* (normal, mild or moderate/severe cognitive problems) as assessed by the Montreal Cognitive Assessment (MoCA) [42] or Mini Mental Status Examination (MMSE) [43].
- *Mobility* as measured by the Timed Up and Go (TuG) [44] and Tinetti-Test [45].
- *Depressive symptoms* according to the Geriatric Depression Scale (GDS) [46].
- *Hand grip strength* in kg as a marker for physical strength.

In addition, the following sociodemographic and health-related parameters will be taken from the patients’ medical records:

- *Age, sex, highest education level, marital state* and living situation/*housing*.
- Use of *aids*, *care* level, *help at home*, *healthcare utilization* (e.g. number of doctor or physician consultations or regular therapy).
- *Diagnoses* and number of *medications*.

These assessments and parameters will serve as independent variables related to self-management. For the assessment of self-management itself and further related factors, the following self-report questionnaires were selected based on the previous literature cited above. The questionnaires will be filled out with the assistance of trained study staff:

- *Self-management* assessed by the Patient Activation Measure (PAM) [47, 48] and Appraisal of Self-Care Agency Scale-Revised (ASAS-R) [49, 50], with the former primarily assessing *confidence* in self-management abilities, and the latter evaluating self-management *behaviour*.
- *Self-Efficacy* as assessed by the generalised self-efficacy scale [51].
- *Anxiety* measured with five items from Becks Anxiety Index (BAI) [52] according to the scale used in the Survey of Health, Aging, and Retirement in Europe (SHARE) [53].
- Quality of Life (*QoL*) as assessed by the short form of the WHO-Quality of Life Questionnaire (WHOQOL-Bref) [54]. This questionnaire also covers satisfaction with social network, mental and physical health, and living environment.
- Health Locus of Control (*HLC*) as assessed by the German Fragebogen zur Erhebung von Kontrollüberzeugungen zu Krankheit und Gesundheit (KKG) [55, 56].
- Views on aging (*VoA*) and subjective age as assessed by the questionnaire used in the German Ageing Survey (Deutscher Alterssurvey, DEAS) [57, 58].

To determine the occurrence and significance of geriatric syndromes, the next step is to utilise a self-created questionnaire using Visual Analogous Scales (VAS). Patients will be requested to select the geriatric syndrome that they are experiencing from a list of options (multiple choice), and to indicate the syndrome that has the greatest impact on their lives (single choice):

- Impaired mobility (walking, stairs).
- Gait problems, falls.
- Cognitive decline/memory problems.
- Depressive symptomology.
- Loneliness.
- Pain.
- Incontinence.
- Sleeping problems.
- Dysphagia.

Patients are then asked to indicate on a scale of 0 to 100,

- how much the respective syndrome affects their daily life,
- whether they would attribute the syndrome to an illness (0) or old age (100),
- how worried they are that the respective syndrome will worsen,
- and how confident they are that the syndrome will improve.

Three and six months after discharge from hospital, all patients will be contacted for a follow-up assessment. For this purpose, patients will be invited to a check-up in an outpatient clinic, where the following assessments will be repeated: Barthel Index [35], MoCA [42], TuG [44] and Tinetti [45], GDS [46], and *hand grip strength*. Additionally, the patients will be asked to answer the following questions: changes in health since the last assessment (same, better, worse, new diagnoses, changes in medication), *healthcare utilization* since the last assessment, changes in *mobility*, changes in concentration, VAS on geriatric syndromes, changes in the syndromes since last assessment (same, better, worse), and *independence* (iADLs and ADLs). Patients will also be asked to re-complete the WHOQOL-Bref [54] and the ASAS-R [49, 50]. Additionally, mortality and reason for death will be noted if necessary.

Those patients unable to come to the outpatient hospital for follow-up screening will be contacted via telephone to complete the additional questionnaires as well as the Barthel Index [35], a measure of *cognition* (immediate and delayed recall and fluency as performed in the MoCA [42]) and the GDS.

The aforementioned data collection will produce both cross-sectional and longitudinal data, enabling a comprehensive understanding of the variables at different time intervals. Separate endpoints are defined for each type of data. For cross-sectional data, the primary endpoint is *self-management*, measured using the ASAS-R [49, 50]. This will help identify factors related to self-management behaviour among older adults, including health-related, psychosocial, and environmental factors.

For longitudinal data, the primary endpoint is *independence* in daily activities. For this purpose, an additive composite endpoint is defined consisting of changes in both iADLs and ADLs. Both ADLs and iADLs are closely related and are expected to change in the same direction, allowing for a combined interpretation [59–61]. These unweighted multi-attribute endpoints assign equal importance to the included instruments and lead to an overall sum score based on all included items [59]. For this purpose, a 1-point change in *independence* is defined as a change of at least 1 point in the Blaylock Score or 5 points in the Barthel Index.

Secondary endpoints for the longitudinal data collection consist of mortality, re-hospitalization, and *QoL*, depending on *self-management*.

### Statistical analyses

The multitude of variables included in the present data collection will allow for an encompassing exploratory and confirmatory analysis of the data. The main goal is to (a) identify the parameters that influence self-management, (b) assess how *self-management* influences future daily *functioning/independence*, and (c) estimate the association between *self-management* abilities and *QoL*. Therefore, as a first step, the *self-management* behaviours of older adults at baseline and their gradient until follow-up will be described. Regression with elastic net regularization [62] or (generalized) linear mixed models (LMMs/GLMMs) will be employed for cross-sectional and longitudinal data to understand how the included health-related, psychosocial, and environmental factors influence self-management as the outcome variable (ASAS-R) [49, 50].

Likewise, mixed models, structural equation (SEM) [63–65] and network analyses [63, 64] will be used to explore the association between *self-management* levels at baseline, and *independence* in ADLs and iADLS at baseline and follow-up. These analyses will furthermore be conducted to understand how baseline *self-management* and related variables influence the *QoL*.

Further exploratory analyses may address the influence of baseline self-management abilities on health outcomes at follow-up, the relative influence of *depressive symptoms* and *VoA* on self-management and health outcomes, and the interactive associations among predictors of self-management as assessed via network analysis or structural equation modelling (SEM).

Similarly, irrespective of self-management, the present data collection will provide a rich understanding of the prevalence and impact of geriatric syndromes in older adults and their impact on *self-management* and ADLs/iADLs.

All analyses will be conducted with a significance level of *p* = 0.05; 95% confidence intervals and effect sizes will be reported, wherever appropriate.

## Discussion

Using a comprehensive, observational, and longitudinal approach, the goals of the planned study are (a) to provide a comprehensive overview of self-management abilities, barriers, and opportunities specific to the older patient population, as they are particularly vulnerable; and (b) to evaluate the impact and prevalence of geriatric syndromes as a potential basis for intervention targets.

As stated in the Background section, there is no universally accepted definition of self-management [16, 19]. For the purpose of this data collection, we thus aim to capture the known predictors and facilitators of self-management in the included questionnaires to confirm their impact on self-management. Self-management in this data collection is preliminarily defined as suggested by The Institute of Medicine based on the framework by Lorig and Holman [66], taking into consideration *all* behaviours performed with the goal to improve health [16]. Those behaviours are assessed by the PAM [47, 48] and ASAS-R [49, 50], as both questionnaires enquire after the general ability to perform “activities” or “measures” concerned with health [47–50]. Previous definitions of self-management postulate that several conditions must be in place to facilitate self-management, such as social support, access to healthcare and information, and appropriate environmental standards [16, 34, 67]. In detail, Lorig and Holman [66] identified five central aspects of self-management, namely active participation, problem-solving and decision-making skills, appropriate usage of available resources, and a sustainable patient-provider relationship. Pearce and Parke [67] provided a more detailed framework, including 14 components describing actions both for the patient and the health care provider, such as information exchange, monitoring, support and access, training, and psychosocial strategies. Other similar self-management concepts include person-oriented attributes, such as active participation, responsibility, and coping with setbacks, as well as person-environment factors, including information regarding the illness, treatment options, individualised care, and reciprocal relationships with health care providers [34]. Udlis [16] identified several necessary self-management steps in concept analysis and concluded that “self-management may occur when the individual has the resources and knowledge needed to adhere to a mutually agreed upon plan while actively participating in the management of their chronic illness”, citing self-efficacy, intention, responsibility, knowledge, resources, and cooperation with health care providers as necessary building blocks of self-management behaviour [16]. All these frameworks highlight the need to not only focus on medical aspects, but also incorporate the biological, psychological, and social aspects of self-management [20].

To confirm the influence of these theoretical factors on patient behaviour, the following parameters are included in the data collection: PAM [47, 48] and ASAS-R [49, 50] cover active participation, knowledge, monitoring of symptoms, and self-efficacy. Additionally, self-efficacy is covered in detail in the Generalized Self-Efficacy Scale [51] and the KKG [55, 56]; as further measures of motivation and activation, mental health is addressed with the BAI and GDS [46, 52]. The WHOQOL-Bref not only provides overall information on *QoL* but also details on living conditions and social networks [54]. This encompassing data collection then serves as a guide for deriving a detailed definition of self-management.

A large variety of self-management endpoints and conceptualizations were used in previous studies [14, 34, 68]. In order to comprehensively evaluate the effectiveness of *self-management*, a thorough understanding of various related factors is needed, such as health and *healthcare utilization*, *QoL*, and daily life *independence*. Although initial findings suggest a correlation between *self-management* and unfavorable health outcomes, it is imperative to identify the most practical and viable endpoint to develop effective clinical interventions [6–8]. Therefore, the current data collection will provide an extensive range of information on health-related and psychosocial variables to determine their connection to self-management.

Finally, it should be noted that several studies and interventions have focused on particular diseases [28–30] and may not be customised to meet the unique needs of the older population. One of the challenges of advanced age is the change in health, social networks, and emotional well-being, making older adults particularly vulnerable [69]. At the same time, advanced age also provides unique skills and resources, such as experience, wisdom, free time, or close family bonds, which should be incorporated as intervention resources [69, 70]. The lifelong learning theory postulates that behavioural changes and adaptation are still possible in older age, although they take longer and are more effortful, thus this particular population has specific needs that many health-behaviour interventions do not take into account [69, 70]. Thus, in the face of demographic change and the high level of healthcare utilization in this population [1, 2], the lack of suitable self-management interventions is a grave oversight. To provide this patient population with suitable interventions, in-depth research on self-management and its accompanying constructs is necessary to facilitate best possible care, reduce healthcare utilization, and maintain independence [16, 19, 34, 67].

### Limitations

The study protocol has certain limitations that should be acknowledged. The primary aim of this study is to assess the factors that affect the ability of elderly patients to independently manage their health. This group of patients is highly vulnerable to multiple health issues, chronic illnesses, and declining health. While this approach may restrict the applicability of the results, it is crucial to improve self-management in this population, as they are the most frequent users of healthcare services. This will not only improve their health and independence but also help healthcare systems deal with demographic changes. Additionally, most questionnaires used in this study rely on self-report. However, this is reasonable since self-management is linked to motivation and each individual’s personal perception of what is achievable [16, 34]. All utilised questionnaires are validated. Finally, although this study protocol describes a multicenter study, it cannot be ruled out that the specifics of the German healthcare system may influence the self-management needs and barriers of the included patients.

### Trial status

Data collection started on 05.04.2023 and is currently ongoing. The study is registered at the German clinical trial register: https://www.drks.de/DRKS00031016.

## Data Availability

The dataset generated in this study will be made freely available for scientific purposes. To obtain the data, please contact the corresponding author.

## Abbreviations

ADLs: Activities of Daily Living
ASAS-R: Appraisal of Self-Care Agency Scale – Revised
BAI: Becks Anxiety Index
DEAS: Deutscher Alterssurvey (German Ageing Survey)
GDS: Geriatric Depression Scale
GLMMs: Generalized Linear Mixed Models
HLC: Health Locus of Control
iADLs: instrumental Activities of Daily Living
MMSE: Mini Mental Status Examination
MoCA: Montreal Cognitive Assessment
PAM: Patient Activation Measure
QoL: Quality of Life
SHARE: Survey of Health, Aging, and Retirement in Europe
SEM: Structural Equation Modelling
TuG: Timed up and Go
VAS: Visual Analogous Scale
VoA: Views on Aging
WHOQOL-Bref: WHO-Quality of Life Questionnaire Short form
